# Supplementary figures and images for: The Effects of Soccer Specific Exercise on Countermovement Jump Performance in Elite Youth Soccer Players
Source: Children (Basel). 2022 Nov 30;9(12):1861. doi: 10.3390/children9121861 (PMC9777183; doi:10.3390/children9121861)

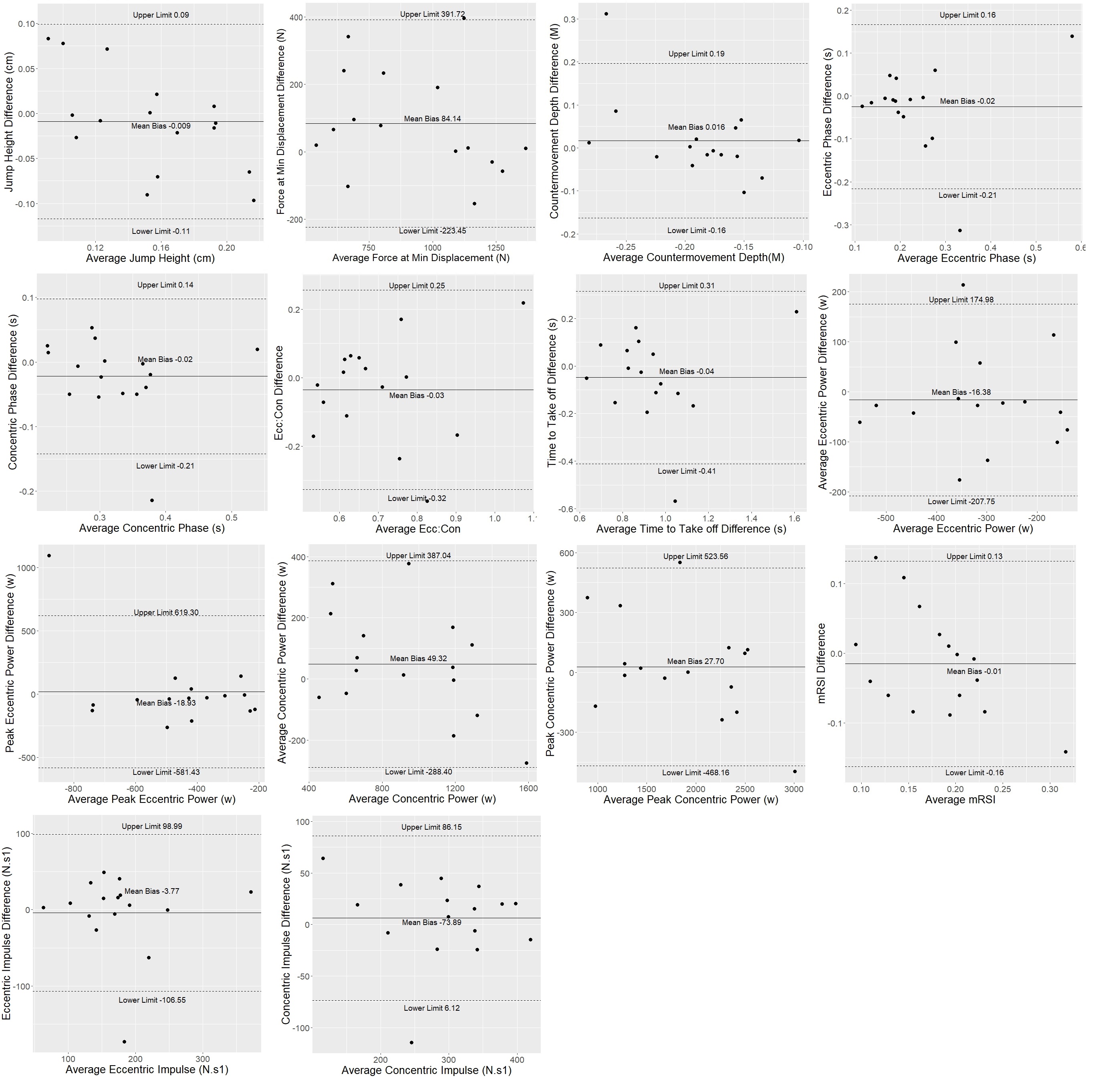

Supplement: Supplementary file 1 [file children-09-01861-s001.zip › Supplementary Files/Supplementary file 3 (CMJND).jpg]

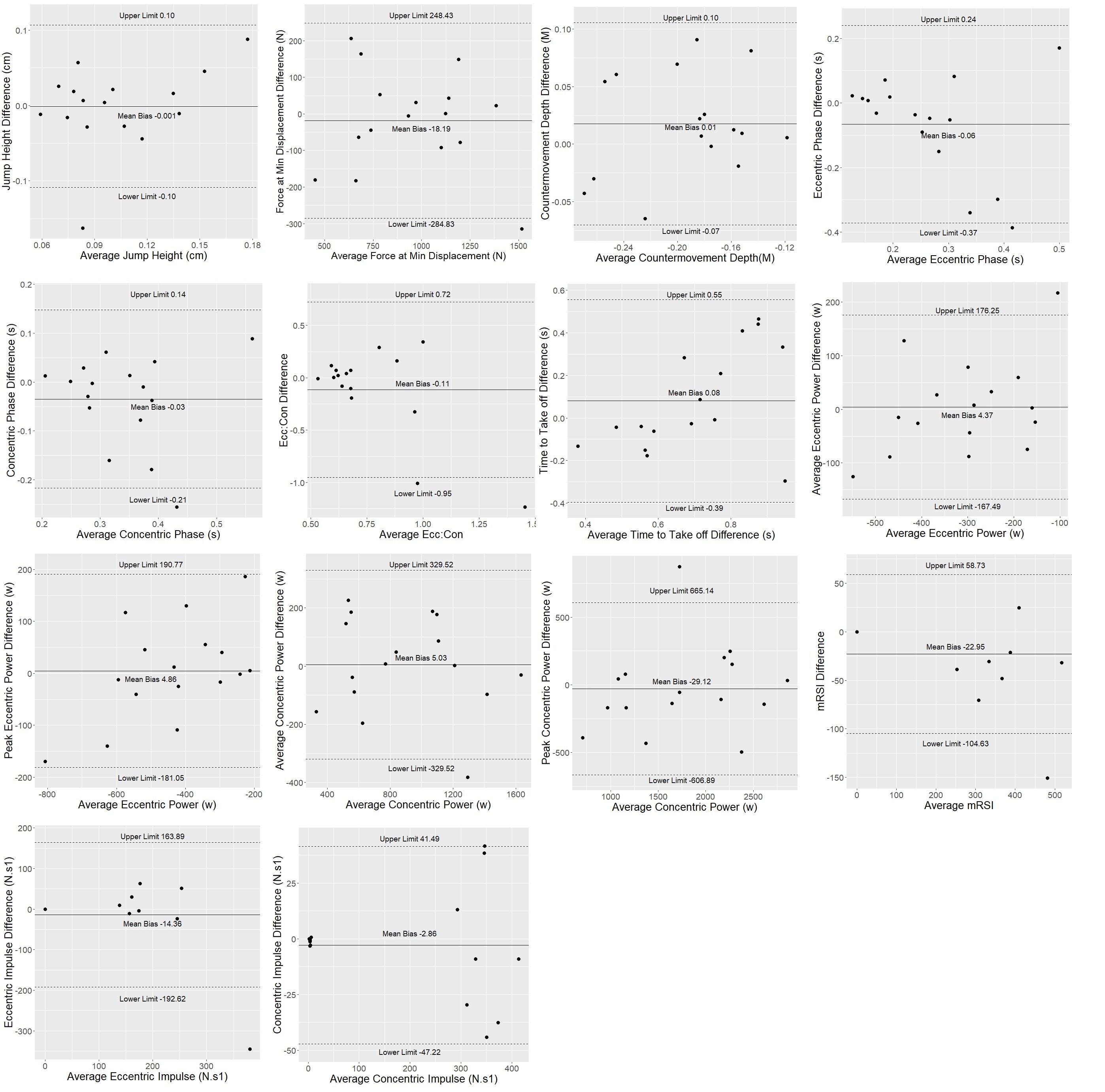

Supplement: Supplementary file 1 [file children-09-01861-s001.zip › Supplementary Files/Supplementary file 2 (CMJDOM).jpg]

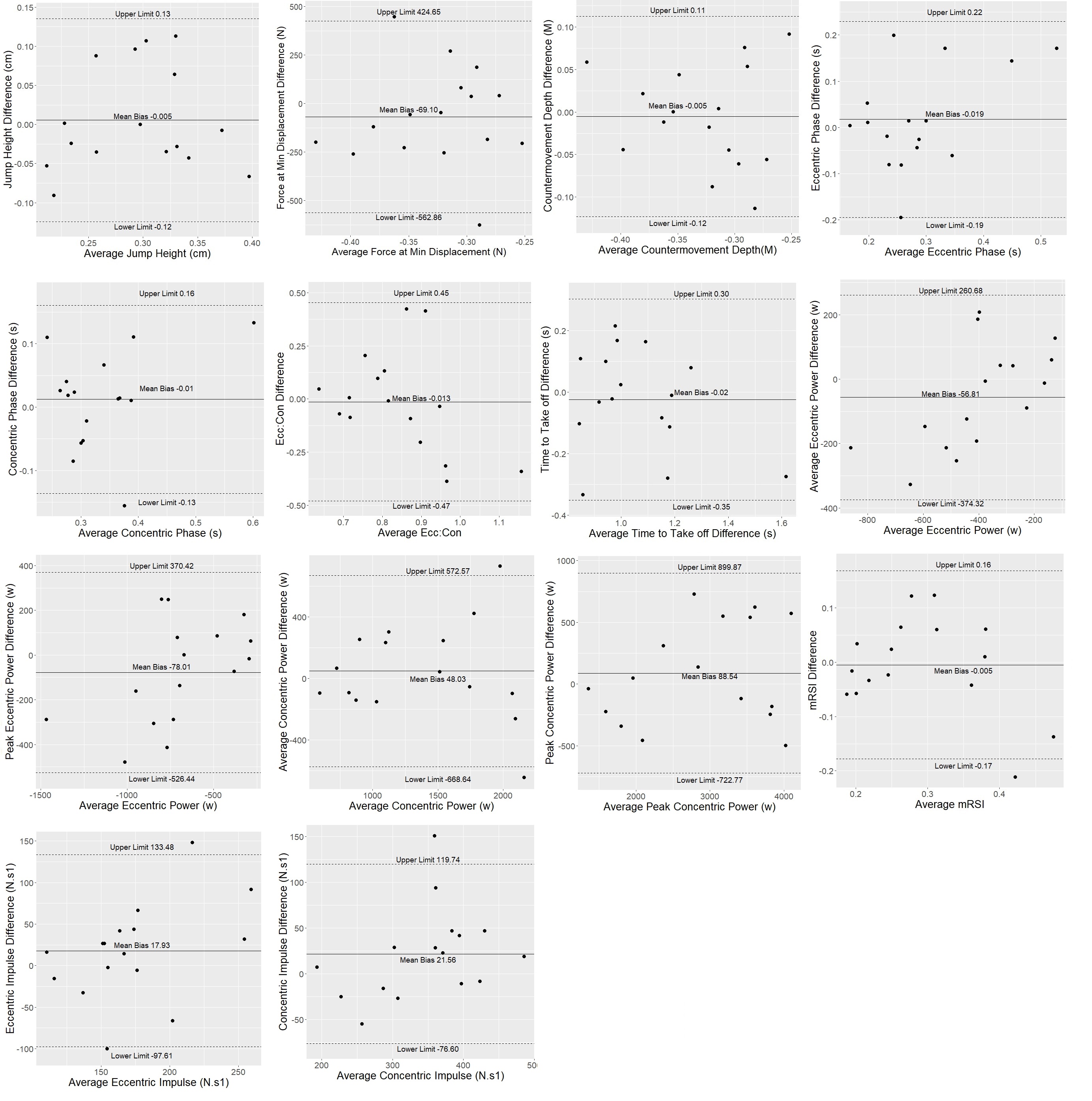

Supplement: Supplementary file 1 [file children-09-01861-s001.zip › Supplementary Files/Supplementary file 1 (CMJBI).jpg]
